# No Consistent Antidepressant Effects of Deep Brain Stimulation of the Bed Nucleus of the Stria Terminalis

**DOI:** 10.3390/brainsci14050499

**Published:** 2024-05-15

**Authors:** Paul B. Fitzgerald, Kate Hoy, Karyn E. Richardson, Kirsten Gainsford, Rebecca Segrave, Sally E. Herring, Zafiris J. Daskalakis, Richard G. Bittar

**Affiliations:** 1 School of Medicine and Psychology, Australian National University, Canberra, ACT 2601, Australia; 2 Bionics Institute of Australia, East Melbourne, Melbourne, VIC 3002, Australia; khoy@bionicsinstitute.org (K.H.); sherring@bionicsinstitute.org (S.E.H.); 3 BrainPark, Turner Institute for Brain and Mental Health, Monash University, Clayton, VIC 3168, Australia; karyn.richardson@monash.edu (K.E.R.);; 4 Monash Biomedical Imaging, Monash University, Clayton, VIC 3168, Australia; 5 Department of Psychiatry, University of California San Diego, La Jolla, CA 92093, USA; 6 Precision Brain Spine and Pain Centre, Melbourne, VIC 3109, Australia; 7 Faculty of Health, Deakin University, Melbourne, VIC 3000, Australia

**Keywords:** deep brain stimulation, major depression, bed nucleus of the stria terminalis, clinical response, treatment resistance

## Abstract

Background: Applying deep brain stimulation (DBS) to several brain regions has been investigated in attempts to treat highly treatment-resistant depression, with variable results. Our initial pilot data suggested that the bed nucleus of the stria terminalis (BNST) could be a promising therapeutic target. Objective: The aim of this study was to gather blinded data exploring the efficacy of applying DBS to the BNST in patients with highly refractory depression. Method: Eight patients with chronic severe treatment-resistant depression underwent DBS to the BNST. A randomised, double-blind crossover study design with fixed stimulation parameters was followed and followed by a period of open-label stimulation. Results: During the double-blind crossover phase, no consistent antidepressant effects were seen with any of the four stimulation parameters applied, and no patients achieved response or remission criteria during the blinded crossover phase or during a subsequent period of three months of blinded stimulation. Stimulation-related side effects, especially agitation, were reported by a number of patients and were reversible with adjustment of the stimulation parameters. Conclusions: The results of this study do not support the application of DBS to the BNST in patients with highly resistant depression or ongoing research utilising stimulation at this brain site. The blocked randomised study design utilising fixed stimulation parameters was poorly tolerated by the participants and does not appear suitable for assessing the efficacy of DBS at this location.

## 1. Introduction

There remains an urgent need for novel and effective antidepressant strategies. Major depressive disorder (MDD) affects a substantial proportion of the population [1], and although a considerable range of antidepressant strategies are available, a significant proportion of patients continue to have inadequate treatment outcomes [2,3]. Deep brain stimulation (DBS) is being increasingly evaluated as a novel method for achieving an antidepressant response in patients who have typically failed to respond to a significant number of traditional antidepressant treatments [4,5].

Approaches investigating DBS in patients with depression have applied stimulation to a number of different brain locations with varying results. The brain regions most commonly targeted have included the subgenual area of the anterior cingulate [6,7], the anterior limb of the internal capsule (ALIC)/ventral striatum (VS) and the nucleus accumbens (NA) [8,9] and the medial forebrain bundle [10,11]. A less commonly targeted site in psychiatric applications of DBS is the bed nucleus of the stria terminalis (BNST). The BNST is a small, multi-component area of the brain situated between the nucleus accumbens and the amygdala, just posterior to the anterior commissure. The BNST is complexly connected to a range of brain regions involved in emotional processing, including the amygdala, hypothalamus and several brainstem nuclei [12]. Studies have indicated that the BNST has a role in the regulation of both mood and anxiety (for example, [13,14]), and there is evidence of disturbed electrophysiological activity in the BNST in patients with MDD. In particular, a study of local field potentials has reported an excess of alpha band oscillations in patients with MDD in the BNST [15]. Both antidepressant and anti-anxiety-like effects have been seen with electrical stimulation of the BNST in animal models [16,17].

Based upon this literature, we initially conducted a small open-label pilot study to explore the antidepressant effects of BNST stimulation in five patients with highly resistant/severe depression [18]. We observed substantial antidepressant efficacy: two patients achieved sustained complete remission of depression, and an additional two patients had lesser but substantial antidepressant effects.

Therefore, the purpose of this study was to further explore the antidepressant efficacy of applying DBS to the BNST and to include a randomised, double-blind evaluation of the antidepressant response. To this end, we conducted a blinded crossover study where the patients received five months of stimulation with five different sets of stimulation parameters, including one month of sham stimulation. Following this, the participants received three months of continued stimulation under blinded conditions using one of the previously utilised settings. We choose to select two variables in the wide range of potential stimulation parameters to evaluate: the electrode locations (either using the two proximal or two distal electrodes) and stimulation voltage (2 or 4 V). This decision was based on our observation in our pilot study that the electrode location and stimulation voltage were far more important to producing clinical benefits than the pulse width or stimulation frequency. From our previous experience, 4 V was the maximum stimulation level that could be initiated without a substantial ramp-up over a number of days or weeks.

## 2. Materials and Methods

### 2.1. Patients

Conducting the study was approved by the Human Research and Ethics Committees of the Epworth, Alfred and Royal Melbourne Hospitals, and the protocol was registered in the Australian and New Zealand Clinical Trials Registry (ACTRN12613000412730). All the patients included in the study were recruited following referral from an external treating psychiatrist.

The study included eight patients, seven female and one male, with a mean age of 49.4 ± 16.1 years.

All of the patients had a diagnosis of MDD as confirmed via assessment by a minimum of three experienced psychiatrists (including the study psychiatrist (P.B.F.)) and the conduct of a Structured Clinical Interview for DSM IV (SCID-II). The patients were required to be experiencing a current episode of depression of at least two years duration and to have had MDD for at least five years. They were also required to have a baseline Montgomery–Åsberg Depression Rating Scale (MADRS) [19] score of more than 25. Extensive past psychiatric history, including the details of all previous psychiatric treatments, was collated from previous treating doctors and hospitals.

Prior to their participation, the patients were presented for surgical approval to the Victorian Mental Health Review Tribunal, which assesses the suitability for DBS under laws restricting access to surgery for psychiatric conditions as a “treatment of last resort”. This review, conducted by a panel including an independent psychiatrist, lawyer and community member, needs to conclude, amongst other considerations, that the patient has the capacity to consent, is providing fully informed consent and has tried all other reasonable treatments for their condition.

For the patients to meet this criterion, they were required to have previously tried all available antidepressant strategies, including adequate trials (at least eight weeks or until not tolerated) of medications from all of the available antidepressant classes (including non-selective monoamine oxidase inhibitors, tricyclic antidepressants, selective serotonin reuptake inhibitors and serotonin noradrenaline reuptake inhibitors, as well as multiple augmentation approaches). They must also have undertaken a minimum of at least two courses of formal structured psychological treatment, including cognitive behavioural therapy, one adequate course of electroconvulsive therapy, and in most cases at least one course of repetitive transcranial magnetic stimulation. Medication was kept stable following DBS surgery and throughout the double-blind phase of the trial. Benzodiazepines or quetiapine were used to treat insomnia as necessary.

Patients were excluded from the study if they had an unstable medical major condition; a neurological disorder; a substantive finding on preoperative medical assessment, including brain MRI scan, or substantial concurrent axis I or II psychiatric comorbidity (a concurrent anxiety disorder or post-traumatic stress disorder was allowed) or were pregnant or lactating.

### 2.2. Operative Procedures

The DBS procedure was conducted using standard means. Electrodes were implanted under local anaesthetic using a stereotactic frame affixed to the patient’s head (Cosman–Roberts–Wells (CRW) frame). With the frame in place, each patient was scanned using CT, which was subsequently fused with a pre-procedural MRI scan for treatment targeting. The DBS electrodes were placed dorso-ventrally down the ALIC to the target site. The target site was 1 mm posterior to the posterior edge of the AC, 4–5 mm lateral to the midline and 2.5–3 mm below the AC–posterior commissure line. This site corresponds to the BNST at the posterior border of the nucleus accumbens, lateral and dorsal to the anterior regions of the hypothalamus. The patients were implanted using Medtronic 3389 electrodes with 1.5 mm contacts and 0.5 mm spacing.

### 2.3. Intra-Operative Stimulation

All the patients underwent intra-operative stimulation from 8 mm above the target (dorsal and lateral) to 2 mm beyond the target (ventral and medial). In each case, the frequency (130 Hz) and pulse width (60 µS) were fixed and the amplitude (current) adjusted progressively up from 1 to 8 mA. Recording of the patient’s subjective responses was carried out at each level over a 30–60 s period of time. 

### 2.4. Post-Operative Stimulation Procedure

Stimulation was commenced a minimum of four weeks following surgery and was planned to progress across three phases.

1. Initial blinded stimulation: This entailed a twenty-week period where the patients were allocated to receive a randomised sequence of five stimulation conditions (listed below) for four weeks at a time. The randomisation was conducted according to a computer-generated random number sequence. The stimulation was programmed by one researcher who was aware of the stimulation type, but the patient and assessor of depressive symptoms were both blind to the treatment condition. The five conditions were:
  - Sham stimulation (all electrodes inactive);
  - Low-intensity “proximal” electrode DBS: This involved stimulation of the two most proximal electrodes on each side at 2 V;
  - Low-intensity “distal” electrode DBS: This involved stimulation of the two distal electrodes on each side at 2 V;
  - Moderate-intensity proximal electrode DBS: This involved stimulation of the two proximal electrodes on each side at 4 V;
  - Moderate-intensity distal electrode DBS: This involved stimulation of the two distal electrodes on each side at 4 V;
2. Blind follow-up phase: During this time, stimulation was recommenced using the stimulation parameters that achieved the maximum reduction in depressive symptoms in phase 1 (active or sham), and these parameters continued for three months. If no substantial anti-depressant response was achieved during phase 1, the patients proceeded to phase 3.
3. Open programming phase: During this phase, stimulation was adjusted on an individualised basis to achieve the optimal clinical response for each patient.

Although the stimulation electrode and voltage varied, as described above, frequency (130 Hz) and pulse width (120 µS) were kept constant through phase 1 and 2.

### 2.5. Assessments

Blinded assessments of depression severity were conducted at initial assessment/pre-surgery, prior to the initiation of the first period of blinded stimulation (baseline), after each four-week period in phase 1, at the end of phase 2 and at 2, 4, 6 and 12 months into phase 3. The primary outcome measure was the MADRS. Data were also collected using the Hamilton Depression Rating Scale (17-item version) (HAMD) [20], Beck Depression Inventory II (BDI) [21] and the Quality of Life Enjoyment and Satisfaction Questionnaire; Short Form (QOL) [22]. The clinician-rated scales (MADRS, HAMD) were administered by two trained and highly experienced research psychologists with established inter-rater reliability (>90%).

All the patients also undertook a battery of cognitive tests at baseline pre-operatively, one-month post-implantation prior to the commencement of stimulation and at six-month follow-up. This included the following tests: Logical Memory I & II (WMS-IV) (contextualised auditory memory), the Controlled Oral Word Association Test (letter fluency; FAS/CFL at alternate assessments), the Rey Auditory Learning Test (non-contextualised auditory memory), the Benton Visuospatial Memory Test (visual memory), the Golden Stroop test (cognitive inhibition), Trail Making Tests A & B, Digit Span Forwards and Backwards (WAIS-III), the Rey Complex Figure test (visuoconstruction, planning and organisation), Verbal Paired Associates (WMS-IV) (associative memory), the Tower of London test (novel problem solving) and Digit Symbol Coding (WAIS-IV) (speed of information processing).

### 2.6. Analysis

Mixed models were calculated to analyse whether there were differences in depressive symptoms (MADRS and HAMD-17 scores) over time. For the analysis of depressive symptoms, we analysed data from the pre-stimulation/post-operative assessment through to the last assessment time point. We used paired *t*-tests to investigate whether there were differences across time points in the group as a whole in the blinded and non-blinded follow-up phases. All the procedures were two-tailed, and significance was set at an α level of 0.05. All the statistical analyses were conducted using SPSS 22.0 (SPSS for Windows. 10.0 Chicago: SPSS; 2013). We had planned to recruit 20 patients for the study. There were no available data from which we thought we could meaningfully estimate the group differences for the blinded controlled phase, and the overall sample size was limited by the practicalities and expense of a single-site DBS trial. We did, however, complete a pre-study power analysis showing that on an open-label basis, we would have had a power of 0.99 to show a 10-point pre-post improvement in the mean group average on the MADRS (SD 9.0, alpha 0.05), which was a lesser effect than that seen in our pilot study [18].

## 3. Results

### 3.1. Participants

Eight patients were recruited, consented and underwent surgery, post-operative stimulation and follow-up (see their clinical characteristics in Table 1). Recruitment ceased after eight patients, as none demonstrated a sustained antidepressant response or remission.

Seven patients were female and one was male, with a mean age of 49.4 ± 16.1 years (see Table 1). The mean age of illness onset was 26.0 ± 15.5 years, and the mean number of depressive episodes was 1.75 ± 0.89. The mean duration of the current illness episode was 12.0 ± 4.8 years. The mean number of failed antidepressant medication trials was 12.4 ± 2.8, and the patients had tried on average 10.9 ± 3.3 other medications for depression. All had been treated with ECT and 7 with rTMS (one had not had rTMS but was undergoing ongoing maintenance ECT prior to DBS). Three patients met the criteria (SCID) for generalised anxiety disorder, two for past PTSD and five for social phobia.

### 3.2. Clinical Outcome

#### 3.2.1. Phase 1: Blinded Crossover

Only three patients completed all four double-blind treatment blocks as per the protocol (see Table 2). Seven completed the sham and one withdrew during this block due to significant deterioration in mood. Seven patients completed the low-intensity proximal electrode 2 mV and distal electrode 2 mV conditions, with one in each group withdrawing due to side effects (agitation/mood deterioration). Moderate-intensity stimulation at 4 mV was not attempted at one electrode when 2 mV stimulation at that electrode produced significant side effects.

**Figure 1:**
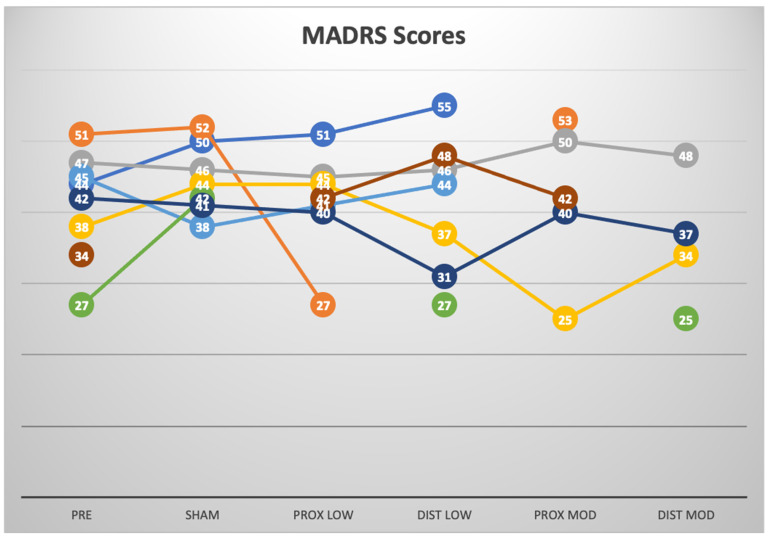
MADRS scores for the double-blind phase.

For the blinded data phase (Table 3, Figure 1), the mixed model analysis of the MADRS scores showed no significant effect of the condition (*p* = 0.73). In addition, there were no significant pairwise comparisons between the mean baseline scores and the mean scores after any of the treatment blocks (proximal 2 V: *p* = 0.9, distal 2 V: *p* = 1.0, proximal 4 V: *p* = 0.84, distal 4 V: *p* = 0.33). There was also no difference in the mean end scores after the sham and any of the other treatment blocks (proximal 2 V: *p* = 0.47, distal 2 V: *p* = 0.43, proximal 4 V: *p* = 0.58, distal 4 V: *p* = 0.10). No differences across blocks were seen in the secondary outcome variables (HAMD data are in Table 2).

No patients met the response or remission criteria at the end of any treatment block.

#### 3.2.2. Phase 2: Blinded Follow-Up

Five patients received three months of blinded continual stimulation based on the “best” stimulation parameters selected from the first phase. For three patients, it was not possible to identify a tolerable and desirable option from phase 1, and as such, they subsequently underwent open-label stimulation only. There were no significant differences in the MADRS or HAMD score from baseline to the end of the three-month blinded follow-up period (MADRS *p* = 0.13, HAMD *p* = 0.13).

**Table 1:** Patient characteristics.

Patient	Age	Sex	Years of Education	Family History of Mood Disorder	Age of Illness Onset	Prior Suicide Attempt/s	Duration of Current Episode (yrs)	Number of Prior Episodes	Number of Antidepressants Trialled	Number of Other Medications (Mood Stabilisers, Antipsychotics, Stimulants)	ECT	rTMS
1	53	F	12	N	45	0	6	1	14	13	>150 across multiple courses	2 courses
2	31	F	13	Y	24	4	7	0	9	8	8 courses	1 course
3	45	F	17	Y	32	0	14	0	13	13	2 courses	1 course
4	45	F	16	Y	22	1	11	2	9	10	Multiple acute and extended maintenance	0
5	26	F	15	Y	10	2	15	0	13	7	>85 across multiple courses	1 course
6	56	F	16	Y	48	0	8	0	10	10	5 courses	1 course
7	66	F	11	N	24	0	20	1	14	9	32 in one extended course of uni and bilateral	Multiple courses
8	73	M	11	Y	3	multiple	15	2	17	17	3 courses	Multiple courses

**Table 2:** Completed and uncompleted blocks of treatment.

Patient	Sham	Proximal 2 mV	Proximal 4 mV	Distal 2 mV	Distal 4 mV
1	completed	completed	stopped	completed	stopped
2	completed	completed	completed	stopped	not attempted
3	completed	completed	completed	completed	completed
4	completed	completed	completed	completed	completed
5	completed	completed	withdrew	completed	stopped
6	completed	Stopped	not attempted	completed	completed
7	completed	completed	completed	completed	completed
8	Withdrew	completed	completed	completed	stopped

#### 3.2.3. Phase 3: Open-Label Treatment

Data were available for seven patients at six-month follow-up and six patients at 12-month follow-up. There were no significant differences in the depression scores over time (MADRS six months, *p* = 0.16, 12 months, *p* = 0.13, HAMD six months, *p* = 0.19, 12 months, *p* = 0.80).

**Table 3:** Patient response.

MADRS									HAMD							
Patient	Pre-Op	Post-Op	Sham	Proximal		Distal		3 Months	Pre-Op	Post-Op	Sham	Proximal		Distal		3 Months
				2 mV	4 mV	2 mV	4 mV					2 mV	4 mV	2 mV	4 mV	
1	43	44	50	51		55			27	24	32	37		34		
2	50	51	52	27	53			29	33	34	35	24	34			25
3	45	47	46	45	50	46	48		29	29	28	25	29	28	27	
4	31	38	44	44	25	37	34	27	25	30	27	26	19	25	26	22
5	43	45	38	41		44		38	29	26	29	25		26		29
6	28	27	42			27	25	28	24	21	33			19	19	20
7	34	42	41	40	40	31	37	38	23	27	28	28	24	22	23	26
8	40	34		42	42	48			27	21		30	30	30		15
Mean	39.3	41.0	44.7	41.4	42.0	41.1	36.0	32.0	27.1	26.5	30.3	27.9	27.2	26.3	23.8	24.4
SD	7.1	7.2	4.6	6.8	9.8	9.2	8.2	4.9	3.0	4.2	2.8	4.2	5.2	4.6	3.1	3.1

### 3.3. Safety and Side Effects

While there were no interoperative complications, one patient underwent follow-up surgery to reposition the battery after it shifted post-surgery. As stated above, a number of patients were unable to tolerate certain fixed double-blind settings. This was, in all cases, due to a combination of agitation and anxiety induced by the onset of stimulation, that in most patients also led to a further lowering of mood. These effects were more commonly experienced for the distal settings (distal 2 mV condition was stopped in one patient and the distal 4 mV condition in three patients). In all cases, the symptoms were transient and resolved with termination of the stimulation settings.

### 3.4. Cognitive Outcome

There were no subjective reports of cognitive impairment, but the cognitive test data were not analysed due to the small sample size (means are presented in Table 4).

## 4. Discussion

No consistent antidepressant effects of BNST DBS were observed in this small blinded study, which was not in line with the results of our prior pilot study. We found no evidence of antidepressant effects across the blinded or open-label follow-up phases, and there were significant issues with the tolerability of initial stimulation that impacted patient retention in each of the blinded phases.

Based on the literature supporting the role of the BNST in mood and anxiety disorders (for example, [14,15,16]) and the promising results of our pilot study [18], where we saw several patients with extremely treatment-resistant depression experience sustained periods of remission, we aimed to gather double-blind data to more robustly demonstrate the antidepressant potential of applying DBS to the BNST. However, we clearly failed to see any persistent or meaningful antidepressant effects with this form of stimulation in the current cohort of patients. Our design was intended to provide a sufficiently long period (four-weeks) of stimulation at each stimulation setting to allow for the emergence of meaningful antidepressant effects, based upon the observation that antidepressant effects of BNST DBS arose relatively quickly following successful changes in the stimulation parameters in our pilot study. We included a subsequent three-month blinded phase, making it unlikely that the lack of an effect was the result of the four-week period being insufficient in duration for some patients for antidepressant effects to emerge.

Blinded stimulation was tolerated poorly in some patients, with problems emerging more commonly at higher voltages and slightly more commonly at the distal electrodes. The low 2 mV and moderate 4 mV levels of stimulation used in the current protocol were relatively modest compared to those required for antidepressant effects in our pilot study. They were selected as we had observed that patients could experience significant over-activation and transient agitation with stimulation of this site if stimulation was commenced at too high a voltage or was not slowly titrated upwards. However, even the relatively modest voltages used in the study were still too high for some patients without a period of upwards titration. While the relatively high level of patient dropout across the treatment blocks has limited the data available for analysis, it is unlikely to have masked a meaningful treatment effect.

DBS is an expensive and complex procedure which has the potential to have life-changing benefits but whose introduction into clinical practice requires thorough evaluation of its efficacy and safety. Because stimulation at some brain sites, including the BNST as we have used, can produce relatively immediate subjective sensations (both physical and emotional), it can be extremely challenging to design studies likely to have an adequate sham/placebo control whilst maximising the likelihood of being able to demonstrate clinical benefits. Randomising patients to receive stimulation at a pre-determined fixed set of stimulation parameters, as we opted to, risks a false negative result if these parameters are suboptimal for individual patients or the implantation site being investigated (or stimulation is provided for too short a period of time to produce the optimal benefits). However, undertaking what can be a substantially lengthy process of establishing the optimal individualised stimulation parameters prior to some form of randomised study phase is highly likely to lead to a lack of successful blinding, where patients become familiar with the sensations associated with stimulation being “on” compared to the sham approach. It is our view that certainly with stimulation applied with standard electrodes to the BNST, it is probably impossible to find an approach that adequately balances these two competing issues and would be a wholly robust test of efficacy. However, the results of the current study suggest that future trials would be better focused on other brain regions, such as the subgenual anterior cingulate or medial forebrain bundle, where there have been recent promising results [23,24].

Clearly, the small sample size of this study is a major limitation on our capacity to generalise from the results, and it is possible that a significantly larger sample may have demonstrated clinical benefits. However, given the invasive nature of DBS surgery and the clear lack of a trend towards benefit, we did not believe that it was justified to continue the study beyond the present sample size.

In conclusion, our study results do not support the antidepressant activity of applying DBS to the region of the BNST posterior to the AC and suggest that other targets are likely to be more fruitful approaches to explore in regard to DBS in the treatment of depression. In this regard, it certainly seems that applying DBS to other brain regions, such as the subgenual anterior cingulate cortex, especially if implantation is guided by appropriate white matter pathway imaging, has greater potential as an antidepressant strategy than stimulation targeted to the BNST [25].

## Figures and Tables

**Table 4:** Cognitive outcomes.

	Baseline			Post Op			Follow Up		
	n	Mean	SD	n	Mean	SD	n	Mean	SD
Logical memory story total immediate recall	8	14.4	7.4	8	18.4	12.2	5	23.0	10.3
Logical memory story total delay recall	8	12.3	9.3	8	12.4	12.5	5	22.0	12.8
Logical memory story total recognition	8	23.1	5.4	8	146.8	344.4	5	25.8	4.7
COWAT score letters	8	33.0	17.7	8	30.5	20.0	6	39.5	14.0
COWAT score animals	8	15.8	8.9	8	15.0	8.1	6	15.7	7.8
RAVLT score trial A1	8	5.3	3.4	8	4.6	2.8	5	6.4	2.2
RAVLT score trial A2	8	8.1	3.5	8	7.4	4.6	5	8.8	2.9
RAVLT score trial A3	8	9.6	3.9	8	133.4	349.8	5	10.6	2.3
RAVLT score trial A4	8	9.1	3.9	8	133.8	349.6	5	11.2	4.0
RAVLT score trial A5	8	10.3	4.3	8	133.8	349.6	5	12.0	3.2
RAVLT score trial B	8	4.5	2.4	8	128.8	351.6	5	5.2	2.2
RAVLT score trial A6	8	8.0	5.2	8	132.3	350.3	5	9.8	5.1
RAVLT score item 7 baseline	8	7.3	5.8	7	8.1	6.2	5	9.8	4.9
RAVLT recognition score	8	12.1	4.1	7	13.4	3.9	5	12.8	3.5
BVMT score trial 1	8	4.6	3.0	8	4.8	4.1	6	5.2	3.9
BVMT score trial 2	8	6.6	3.1	8	7.1	2.9	6	7.5	3.7
BVMT score trial 3	8	7.4	3.2	8	8.5	3.1	6	8.7	3.4
BVMT score delay	8	6.8	3.8	8	8.1	4.1	6	9.0	3.5
BVMT score recognition	8	11.0	0.9	8	11.3	1.0	6	11.2	1.0
Stroop word score	8	75.5	29.2	6	74.5	15.9	5	77.2	22.8
Stroop colour score	8	51.8	17.4	6	56.8	12.6	5	60.8	13.0
Stroop word-colour score	8	23.5	12.6	6	32.2	14.4	5	37.6	8.7
Stroop interference	4	−5.2	5.4	3	−5.5	8.5	0		
Trail-making A time	8	41.9	22.5	8	44.0	33.2	6	43.3	30.7
Trail-making B time	6	111.0	39.6	6	91.3	41.5	6	111.8	60.0
Digit span forward score	8	7.8	3.3	8	8.4	4.0	5	8.2	3.2
Digit span forward longest number score	8	5.3	1.6	8	5.1	1.8	5	5.6	1.8
Digit span back score	8	7.4	2.4	8	6.9	3.3	5	7.2	3.4
Digit span back longest number score	8	4.0	1.2	8	3.8	1.6	5	4.0	1.9
Digit span sequence score	8	7.0	1.8	8	6.3	2.4	5	7.4	2.8
Digit span sequence longest number score	8	7.9	7.2	8	4.5	1.3	5	5.2	1.1
Digit span total	8	21.8	7.2	8	20.6	9.8	5	22.8	8.6
RCFT copy score	8	32.3	5.6	6	30.8	6.2	5	32.4	5.0
RCFT delay recall score	8	12.3	8.5	6	178.9	401.9	5	16.5	10.0
Verbal paired associates—immediate	6	34.7	14.2	5	229.2	430.6	5	41.4	15.1
Verbal paired associates—delay	6	9.2	4.2	5	207.6	442.4	5	11.0	3.7
Verbal paired associates—recognition	6	32.8	9.7	5	229.8	430.0	5	37.6	2.9
Coding at baseline	8	50.0	13.4	6	49.5	17.3	4	53.8	19.7
Tower of London—total correct	5	7.0	3.4	6	170.5	405.9	4	6.0	3.2
Tower of London—total move score	7	38.1	18.5	6	204.3	389.6	4	41.3	14.9

COWAT = Oral Word Association Test, RAVLT = Rey Auditory Learning Test, BVMT = Benton Visuospatial Memory Test, RCFT = Rey Complex Figure test, SD = Standard deviation.

## Data Availability

Data is not publicly available as sharing of data from this study is restricted by ethics approval and privacy regulations.
